# Strategies to Improve the Quality and Freshness of Human Bone Marrow-Derived Mesenchymal Stem Cells for Neurological Diseases

**DOI:** 10.1155/2021/8444599

**Published:** 2021-09-08

**Authors:** Da Yeon Lee, Sung Eun Lee, Do Hyeon Kwon, Saraswathy Nithiyanandam, Mi Ha Lee, Ji Su Hwang, Shaherin Basith, Jung Hwan Ahn, Tae Hwan Shin, Gwang Lee

**Affiliations:** ^1^Department of Physiology, Ajou University School of Medicine, Suwon, Republic of Korea; ^2^Department of Emergency Medicine, Ajou University School of Medicine, Suwon, Republic of Korea; ^3^Department of Molecular Science and Technology, Ajou University, Suwon, Republic of Korea

## Abstract

Human bone marrow-derived mesenchymal stem cells (hBM-MSCs) have been studied for their application to manage various neurological diseases, owing to their anti-inflammatory, immunomodulatory, paracrine, and antiapoptotic ability, as well as their homing capacity to specific regions of brain injury. Among mesenchymal stem cells, such as BM-MSCs, adipose-derived MSCs, and umbilical cord MSCs, BM-MSCs have many merits as cell therapeutic agents based on their widespread availability and relatively easy attainability and in vitro handling. For stem cell-based therapy with BM-MSCs, it is essential to perform ex vivo expansion as low numbers of MSCs are obtained in bone marrow aspirates. Depending on timing, before hBM-MSC transplantation into patients, after detaching them from the culture dish, cell viability, deformability, cell size, and membrane fluidity are decreased, whereas reactive oxygen species generation, lipid peroxidation, and cytosolic vacuoles are increased. Thus, the quality and freshness of hBM-MSCs decrease over time after detachment from the culture dish. Especially, for neurological disease cell therapy, the deformability of BM-MSCs is particularly important in the brain for the development of microvessels. As studies on the traditional characteristics of hBM-MSCs before transplantation into the brain are very limited, omics and machine learning approaches are needed to evaluate cell conditions with indepth and comprehensive analyses. Here, we provide an overview of hBM-MSCs, the application of these cells to various neurological diseases, and improvements in their quality and freshness based on integrated omics after detachment from the culture dish for successful cell therapy.

## 1. Introduction

Bone marrow-derived mesenchymal stem cells (BM-MSCs) have many merits as cell therapeutic agents, such as comparably easy in vitro handling, high plasticity, widespread availability, and immunosuppressive activity [1–3]. In addition, they have beneficial characteristics, such as anti-inflammatory, immunomodulatory, paracrine, and antiapoptotic ability, as well as homing capacity to the region of brain injury. Particularly, BM-MSCs can suppress inflammatory conditions in the central nervous system (CNS) and home to inflammatory brain injury [4–9]. To date, there have been many drugs developed to reduce the symptoms of CNS diseases because of irreversible neurological damage and limited regeneration in the brain, but these are associated with many adverse effects [10–12]. Therefore, BM-MSCs are a promising approach to treat neurological diseases, such as ischemia, traumatic brain injury, and neurodegenerative diseases, owing to their anti-inflammatory and immunomodulatory effects on such CNS neurological diseases [3, 13–16].

**Figure 1:**
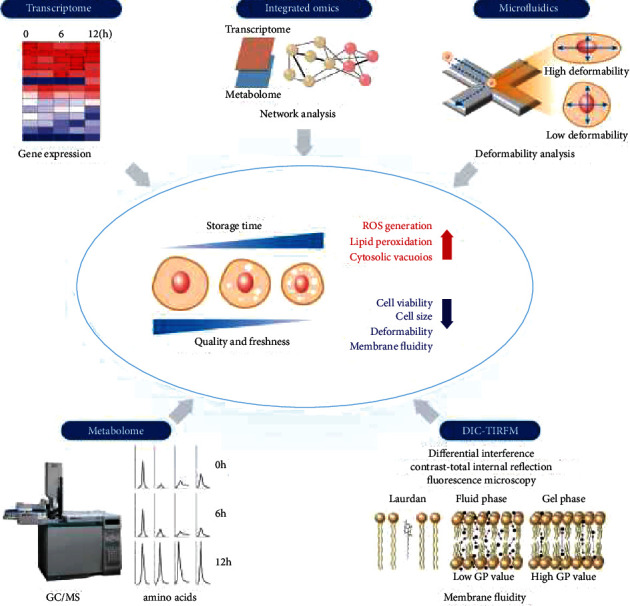
The quality and freshness of human bone marrow-derived mesenchymal stem cells (hBM-MSCs) are decreased over time after detachment from the culture dish. To evaluate the freshness and quality of hBM-MSCs, the metabolome is analyzed using gas chromatography–mass spectrometry (GC/MS), the transcriptome is analyzed using microarray, deformability is analyzed using microfluidics, and membrane fluidity is tested using differential interference contrast- (DIC-) total internal reflection fluorescence microscopy (TIRFM) in combination based on previous reports [9, 24].

Most studies on BM-MSC-based therapies for neurological diseases have focused on the paracrine effects, immunomodulatory effects, and neuronal replacement through differentiation [17–19]. In addition, the MSC-based cell therapies have been applied to neurological diseases, which have no effective alternative treatments. Andrzejewska et al. summarized the application of MSC on the neurological diseases, including stroke, brain injury, Alzheimer's disease (AD), Huntington's disease (HD), Parkinson's disease (PD), amyotrophic lateral sclerosis (ALS), multiple sclerosis (MS), and spinal cord injury, with experimental and clinical aspect [20]. Mukai et al. focused on clinical trial of the MSC transplantation in the neurological diseases with detailed condition of the clinical trials [21]. Moreover, Namestnikova et al. reported advantages of combinatorial methods, which are combination of coadministration of different stem/progenitor cell types, for the neurological diseases in animal and clinical study [22]. However, studies on the characteristics of human (hBM-MSCs) before transplantation are very limited. Depending on timing, before hBM-MSC transplantation into patients, after detaching these cells from the culture dish, cell viability, deformability, cell size, and membrane fluidity decrease, whereas reactive oxygen species (ROS) generation, lipid peroxidation, and cytosolic vacuoles increase, as shown in Figure 1 [9, 23].

hBM-MSC transplantation into patients is associated with an inevitable time-delay after cell detachment from the culture dish owing to various factors, including the injection formulation, transportation, and surgery preparation. Thus, an assessment of the quality and freshness of hBM-MSCs is important for successful hBM-MSC-based cytotherapy outcomes, and studies have tried to evaluate and preserve the quality and freshness of hBM-MSCs [23, 25]. However, conventional cell-based methods for evaluation, such as cell viability assays, fluorescence-activated cell sorting-based methods, and ultrastructural analysis, do not reveal the mechanism underlying changes in the quality and freshness of hBM-MSCs. Thus, omics, including genomics, transcriptomics, proteomics, and metabolomics, yield comprehensive information and can be interpreted using bioinformatic analysis [24, 26–31]. These technologies have been introduced to analyze the mechanism of changes in the hBM-MSCs status [9, 23]. Especially, the integration of transcriptomics and metabolomics with amino acid profiles is helpful to elucidate the quality and freshness of hBM-MSCs over time after trypsinizing cells [23]. Recently, advanced analysis has been used for the integration of omics, identification and in silico prediction of biological functions, and screening of upstream regulator molecules [32]. Moreover, discrimination methods, such as machine learning algorithms, have been used for investigations of correlations among each omics dataset, based on the large amount of data acquired from multiomic analysis. To evaluate and maintain the quality and freshness of hBM-MSCs, comprehensive multiomic analysis (big data) and proper machine learning algorithms for analyses of correlations within data are highly recommended rather than target approaches, according to the complexity of cellular changes after detachment from the culture dish. Here, we review three topics as follows: (i) hBM-MSCs, (ii) the application of hBM-MSCs to various neurological diseases, and (iii) the improvement of the quality and freshness of BM-MSCs after detachment from the culture dish for successful cell therapy.

## 2. hBM-MSCs

MSCs were first discovered in the bone marrow by Friedenstein in the 1970s [33, 34]. These cells are nonhematopoietic multipotent adult stem cells that are plastic-adherent with great capacity for proliferation, self-renewal, and differentiation [35, 36]. MSCs can be obtained not only from bone marrow but also from various tissues, such as adipose tissue, placenta, umbilical cord, and peripheral blood [37–40]. Although MSCs can be of different tissue origins, they must meet the minimal criteria proposed by the Mesenchymal and Tissue Stem Cell Committee of the International Society for Cellular Therapy (ISCT) as follows: (1) maintenance of plastic adherence; (2) ≥95% of the MSCs express surface molecules, such as CD73, CD90, and CD105, and do not express surface molecules, such as CD19 and HLA class I or CD11b, CD79a or CD45, CD34, and CD14; (3) capacity of trilineage differentiation in vitro into adipocytes (fat), osteoblasts (bone), and chondrocytes (cartilage); and (4) immunomodulatory activity [17, 41–46]. Additionally, MSCs can be differentiated into nonmesodermal-origin cells, including neurons, hepatocytes, cardiomyocytes, hepatocytes, and epithelial cells, which are of ectodermal and endodermal lineages [47–51].

Among MSCs, BM-MSC-based therapies have been promising strategy in preclinical and clinical trials based on tissue regeneration and wound healing attributed to the cell engraftment and differentiation properties of MSC [45, 52–55]. However, recent approaches for BM-MSC therapies have focused on paracrine effects in which MSC-derived vesicles are secreted containing various contents, such as soluble cytokines, growth factors, hormones, and miRNA, from immune cells and damaged tissues. This effect finally improves the efficacy of BM-MSC therapy [56–59]. As many studies have been reported regarding the efficacy of using exosomes derived from BM-MSCs on diverse diseases [59, 60], such BM-MSC-based therapies have been continuously suggesting to be promising strategies for clinical application to various neurological diseases [16, 20, 61, 62]. In addition, obtaining hBM-MSCs from adult tissue can avoid controversy regarding the ethical issues associated with the use of embryonic sources [63, 64]. Owing to these advantages, hBM-MSCs have strong potential in neurological diseases as a therapeutic tool.

## 3. Application of hBM-MSCs in Neurological Diseases

Neurological diseases, which cause neurological impairment, are characterized by irreversibility and progressive disorders, resulting in deterioration of the performance of regular activities because of the limited regenerative capacity for lost neurons and glial cells [16, 20, 65]. However, the landscape of treatment is limited, with restricted treatment options [16, 65]. Stem cell therapy, from preclinical to clinical trials based on the fundamental characteristics of stem cells, has shown promise as a potential treatment or to at least prevent progressive deterioration with neurological diseases, spinal cord injury, and myocardial infarction [65–67]. Several different sources, including neural stem cells, human umbilical cord blood cells, embryonic stem cells, hematopoietic stem cells, and MSCs, have been utilized in stem cell therapy [65]. Neural stem and embryonic stem cells have not been easy to apply in clinical fields or research because of the ethical issues (procured from aborted fetuses for allogenic transplantation), allograft rejection, or tumorigenic capacity [65, 68]. In recent years, more than half of registered stem cell trials have been conducted using MSCs because they are easy to acquire from the patients themselves, avoiding the ethical concerns and the possibility of harmful events [14, 65, 68]. In this section, we briefly review the neuroprotective and anti-inflammatory effects of hBM-MSCs via systemic transplantation, such as intravenous or intraarterial infusion, as shown in preclinical and clinical studies on ischemic stroke, traumatic brain hemorrhage, and neurodegenerative diseases, such as AD, HD, and PD.

### 3.1. Ischemic Stroke

The transplantation of hBM-MSCs could improve functional recovery and reduce the infarction size via neuroprotective and immunomodulatory effects after ischemic stroke in rats [69–74], a nonhuman primate model [75], and humans [14, 76–81]. Neuroprotection, nerve regeneration, and angiogenesis result from the paracrine effect of neurotrophic factors, including brain-derived neurotrophic factor (BDNF), nerve growth factor (NGF), and vascular endothelial growth factor (VEGF) [74, 82]. The local activation of astrocytes and microglia/macrophages and the influx of leukocytes, including T cytotoxic cells, are significantly reduced [60]. Immunomodulatory effects as suppressors of inflammation were observed by decreasing the levels of proinflammatory cytokines, namely, interleukin (IL)-1*α*, IL-1𝛽, IL-6, and tumor necrosis factor (TNF)-*α* and by increasing the levels of anti-inflammatory cytokines, including IL-4, IL-10, and interferon (INF)-𝛽 [60, 83, 84]. Moreover, rat BM-MSCs can suppress the inflammatory response by decreasing activated microglia, which are resident immune cells of the brain that produce proinflammatory cytokines at the cellular network level [85]. Interestingly, in an ischemic stroke animal model, hBM-MSCs were found to restore polyamine and free fatty acid compositions from metabolic disturbance to a near-normal state and maintain metabolic homeostasis [86, 87]. Migrated leucocytes aggravate neuroinflammation, enhancing cell death, blood–brain barrier (BBB) disruption, and vasogenic edema [71, 88, 89]. Leucocyte infiltration is facilitated by an increase in BBB permeability and endothelial cell adhesion molecule expression [89]. Leucocytes further enhance inflammation, increase cell death, and lead to BBB disruption and vasogenic edema [89]. Particularly, hBM-MSCs can decrease BBB permeability in the damaged neural tissue [71] and provide BBB integrity and maintenance through interactions with pericytes, astrocytes, and neurons [88].

An initial clinical trial of hBM-MSCs using autologous stem cells was conducted in 2005 for five patients with midcerebral artery occlusion, comparing results to those of twenty-five patients without stem cell therapy [14]. To date, phase I/II studies, including the first study, have reported the safety and feasibility of autologous or allogenic hBM-MSCs with long-lasting or transient neurological improvements [14, 76–81], functional improvements [80], and a short-term decrease in circulating T cells and inflammatory cytokines [90].

Although serious complications of hBM-MSCs have not been reported, there are some concerns about events such as pulmonary embolism with the intravenous injection of adipose MSCs [91] and allogeneic BM-MSCs [92], as well as the possibility of microembolism risk due to the closure of the lumens of small-diameter vessels related to the flow of cerebral blood, cell dose, infusion velocity [92], and innate procoagulant activity [67]. In addition, a previous study revealed that the deformability of hBM-MSCs decreased, and that the membranes of hBM-MSCs became stiffer via the peroxidation of plasma membrane lipids over time owing to the generation of ROS [23]. Cell dose and infusion velocity are important factors that trigger embolism; however, changing the decreased deformability of hBM-MSCs is an important contributing factor to these serious complications, considering cerebral infarction in patients with sickle-cell disease, which decreases the deformability of red blood cells [93]. Therefore, it is necessary to fully consider the quality and freshness of hBM-MSCs after dissociation from the cell culture dish.

### 3.2. hBM-MSCs in Traumatic Brain Injury

Traumatic brain injury is caused by primary injury facilitated by an initial insult and secondary injury occurring 1–3 days after the initial traumatic event. Primary injury includes a direct response to the initial insult, such as BBB disruption, cranial hemorrhage, brain swelling, and an acute reaction mediated by oxidative stress and excitotoxicity [94, 95]. Secondary injury is associated with the release of excitatory amino acids, ionic imbalances, intracellular calcium overload, mitochondrial dysfunction, and several immunological and inflammatory responses. This reaction induces ongoing neurodegeneration, diminished neurogenesis, axonal damage, and cell death [96, 97]. Since the complexity of injury-associated mechanisms has led to the need for multi-target treatment, several studies have been conducted using MSCs with various paracrine activities. In an animal model, subjects treated with rat BM-MSCs showed attenuated motor and cognitive deficits through the induction of trophic factors, such as BDNF and NGF, which promoted neurogenesis, neuroprotection, neural repair, immunomodulatory activity, and the secretion of bioactive factors, such as exosomes [15, 98, 99]. Some other studies using hBM-MSCs also showed functional improvements with immunomodulatory activity and the secretion of bioactive factors, such as exosomes [100, 101].

Several clinical studies have been conducted based on preclinical study results [102]. Cox et al. conducted intravenous injections of human BM-mononuclear cells in 25 patients after severe traumatic brain injury. Based on the results, there were no serious adverse events and the preservation of functionally critical regions, and the downregulation of inflammatory cytokines was observed [103]. Tian et al. injected autologous hBM-MSCs via lumbar puncture into 97 patients with subacute-stage traumatic brain injury and showed the safety and effectiveness of this therapy [104]. Zhang et al. conducted topical injection to the injured area using autologous hBM-MSCs and also showed the safety and feasibility of cell therapy [105].

### 3.3. hBM-MSCs in Neurodegenerative Disease

Neurodegenerative disease initially damages various types of neurons or glial cells but ultimately specifically causes the loss of function of certain cells, such as hippocampus and frontal lobe dysfunction in AD, striatal dopaminergic neurons in PD, or dysfunction of the striatal structure in HD. Although there are treatments to relieve symptoms for some neurodegenerative diseases, no treatments have been found that can modify the disease course [16, 20]. From this point of view, many studies have been conducted using BM-MSCs, which have the potential to replace lost cells and functional restoration through various paracrine activities [16, 20, 65].

### 3.4. AD

AD is a clinical dementia-presenting disease, and neuroinflammation mediated by the accumulation of amyloid beta plaques and neurofibrillary tangles is known as the main pathological mechanism [106, 107]. Based on this pathophysiology, several studies have been conducted using MSCs. In animal models, mouse or rat BM-MSC infusion improved cognitive impairment through various mechanisms, such as enhancing neurogenesis in the hippocampus [108, 109], increasing the level of acetylcholine [110], stabilizing and regenerating the synapse [111, 112], and modulating immunomodulatory activity [113]. Studies using hBM-MSCs have also shown reduced amyloid beta deposition [114, 115] and increased amyloid beta clearance [116] and neurogenesis [117].

Based on animal research, several clinical trials are ongoing [20, 118]. Initial clinical trials using human umbilical cord or umbilical cord blood-derived MSCs (NCT01547689, NCT01696591, and NCT02054208) showed safety, but no positive results have been reported to improve the clinical status of AD patients. In addition, similar clinical trials are ongoing in several countries [20]. Although there have not been many clinical trials using hBM-MSCs, if the major pathophysiology of AD is associated with neuroinflammation, hBM-MSC therapy with paracrine effects might still be a promising treatment option [118, 119]. In the future, we expect that research using the replacement potential of BM-MSCs or that using BM-MSCs for early-stage AD will be performed.

### 3.5. PD

PD is a disease characterized by a gradual decrease in dopamine-producing neuronal cell in the *substantia nigra* and is accompanied by alpha synucleinopathy that results in the formation of Lewi bodies [120]. In PD, there are treatments to improve symptoms but no treatment options for the disease itself. For these reasons, therapy using MSCs in a PD animal model has been attempted. In experimental studies, rat BM-MSC administration has resulted in improvements in motor functions in PD animal models [121, 122], and other studies have shown that these results are associated with elevated dopamine levels in the striatum, enhanced neurogenesis, inhibited transmission of alpha-synuclein, and immunomodulatory effects [122–125]. A study has also shown that the preconditioning of BM-MSCs is more effective [126].

In clinical trials, the safety of hBM-MSC therapy was established in studies of transplantation through the stereotactic surgical method and intra-arterial administration using the cerebral artery, and improvements in motor function were observed in some patients [62, 127]. Currently, a phase II study is also being conducted for patients with idiopathic PD (NCT04506073). As results of previous experimental studies and preliminary data from clinical trials have shown that hBM-MSC treatment is safe and helpful in improving motor function, therapy using hBM-MSCs has the potential to comprise a disease-modifying treatment for PD patients.

### 3.6. HD

HD is a rare genetic disorder that causes cognitive impairment and movement abnormalities due to a mutation in the gene encoding the protein huntingtin, followed by damage to the striatal structure secreting gamma aminobutyric acid [128]. Effective treatment for HD has not been found. In an animal model, BM-MSC injection was mainly performed intracerebrally due to the selective damage to this area in HD. Transplanted rat or mouse BM-MSCs has been shown to activate endogenous neural stem cell proliferation and reduce apoptotic cell death through increases in BDNF or NGF levels in the striatal area, and as a result, the motor and memory function of the HD-model mice treated with MSCs were improved [129, 130]. Even with intranasal administration, an HD mouse model treated with mouse BM-MSCs showed an improved sleep cycle and survival time mediated by an increase in striatal expression of the factor associated with dopamine receptor protein and an immunomodulatory effect [131].

Based on these experimental studies, therapy with MSCs has been considered a potential disease-modifying treatment option for patients with HD, like that for other neurodegenerative diseases [132], but clinical trials have not yet been actively conducted. Zuccato et al. have reported low BDNF levels in HD patients, and that these low levels, considered one component of disease pathophysiology, are less useful as a biomarker of disease onset and progression in HD patients [133]. Owing to the complexity of symptomatology and pathophysiology, there have been observational clinical trials performed to clarify the clinical symptoms and detect potential therapeutic targets before cell therapy (NCT01937923). However, no positive results in humans have been found to date. Thus, BM-MSC treatment will be a meaningful potential treatment for HD patients, as previous experimental studies have shown that this approach improves functional activity and reduces brain atrophy.

### 3.7. Improvements in the Quality and Freshness of BM-MSCs after Detachment from the Culture Dish

**Figure 2:**
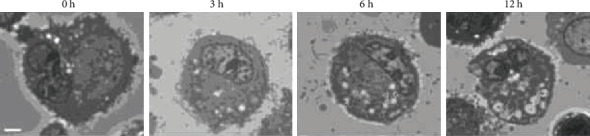
Representative images (×1,000) of starved human bone marrow-derived mesenchymal stem cells (hBM-MSCs). Cells were starved in phosphate-buffered saline at room temperature for 0–12 h in a previous report [9]. Scale bar = 2 *μ*m.

Although hBM-MSCs are considered a potential therapeutic tool for various neurological diseases, a major bottleneck in the clinical application of hBM-MSCs is maintaining individual stem cell properties during ex vivo expansion, which is essential to achieve a therapeutically relevant number of cells. This is because only 0.001–0.01% of cells in the bone marrow are mononuclear cells [134]. After the expansion process, hBM-MSCs are detached from the culture dish and subjected to a serum-starved condition, which is largely different than their original environment, such as the MSC niche, and cells lose their useful properties [135–138]. Previous reports evaluated the freshness of hBM-MSCs kept in phosphate-buffered saline (PBS) over time after trypsinization, which can mimic ex vivo storage conditions [9, 23]. The cell viability was decreased through membrane peroxidation, and the number of cytosolic vacuoles was increased, depending on the PBS storage time, as shown in Figure 2 [9]. In addition, the expression levels of apoptosis and stress-related genes were significantly increased in hBM-MSCs after detachment from the culture dish over time [9, 23]. As hBM-MSCs are sensitive to microenvironmental conditions, stem cells stored in long holding induced cell aggregation and affected the differentiation potential of hBM-MSCs [23, 139, 140]. Therefore, hBM-MSCs should be transplanted as soon as possible after detachment from the culture dish. Even though the quality and freshness of hBM-MSCs is highly dependent on the preparation of cells and manufacturing practices, we have previously shown that the maximum storage time for optimal transplantation is within 6 h because profiles for transmission electron microscopy (TEM) imaging, gene expression, deformation index, storage time, cell viability, and metabolism are altered after storing cells for 6 h in holding conditions in the hBM-MSC group compared to the control group (0 holding stored hBM-MSC group) [9, 23].

The quality and freshness of hBM-MSCs after detachment from the culture dish were also previously analyzed with respect to viability, ultrastructure, deformability, cellular size, membrane fluidity, transcriptomics, and metabolomics [9, 23]. Cell deformability reflects the physicochemical changes in cellular components, such as nuclear organization, the cytoskeleton, and the membrane, in microfluidic devices [141]. For example, the deformability of red blood cells (RBCs) in diabetes, sickle-cell disease, and malaria is reduced, compared to that of healthy RBCs [142]. This is one reason as to why oxidative stress and lipid peroxidation reduce the deformability of RBCs [143]. It was reported that hBM-MSC deformability, used to analyze the quality and freshness of hBM-MSCs based on measurements using microfluidic devices, was slightly decreased after 12 h of storage [23] but was significantly reduced after 24 h of storage in PBS [144]. These results also suggested a decrease in cell deformability and membrane fluidity mediated by ROS generation and lipid peroxidation over storage time after cell detachment [23]. Therefore, these data suggest that for hBM-MSC-based cell therapy for neurological diseases, cell deformability in the brain with developing microvessels is one key point that should be considered.

**Figure 3:**
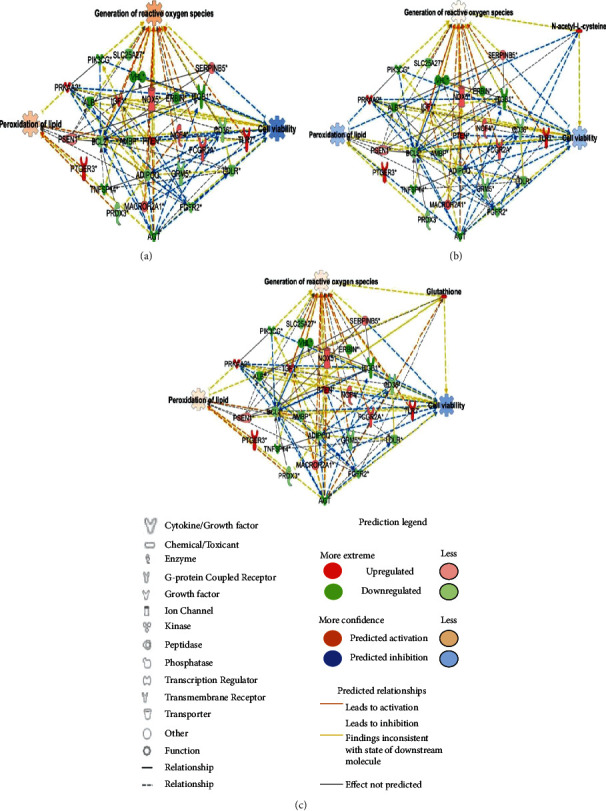
Transcriptomic network related to the quality and freshness of starved human bone marrow-derived mesenchymal stem cells (hBM-MSCs). (a) Analysis of the transcriptomic network with prediction using Ingenuity Pathway Analysis based on starved hBM-MSCs in phosphate-buffered saline for 12 h. Analysis of the transcriptomic network based on a combination of (b) N-acetyl-L-cysteine (NAC) and (c) glutathione (GSH) administered for 12 h. The analysis involved a fold change cut − off value ± 9. Green and red nodes indicate genes that were up and downregulated, respectively, compared to control levels. Orange and blue arrows indicate in silico prediction of function as activation and inhibition, respectively. Details of the shape and color, which were created with Ingenuity Systems (http://www.ingenuity.com), are described in the legends.

An analysis of genes related to the quality and freshness of starved-hBM-MSCs for 6 and 12 h in PBS showed 27 genes that were changed, when compared to their levels in control hBM-MSCs (Table 1) based on previous reports [9, 23]. Compared to that after storage for 6 h, the gene expression was highly altered by storage for 12 h. Thus, we analyzed the transcriptomes of hBM-MSCs after 12 h based on three main functions, the generation of reactive oxygen, lipid peroxidation, and cell viability. The transcriptomic network related to each function in hBM-MSCs stored for 12 h was connected, and the functions were algorithmically predicted using Ingenuity Pathway Analysis. This in silico prediction indicated that ROS generation and lipid peroxidation were increased, and cell viability was decreased (Figure 3(a)). These data suggested that regulating redox homeostasis will be one key point to keep hBM-MSCs healthy and fresh in the pretransplantation stage.

Antioxidants can be used to eliminate ROS production. Accumulating studies have found that antioxidants can decrease the toxicity of ROS, including superoxide dismutase, glutathione (GSH), peroxidase, and vitamin E [145, 146]. To evaluate the effect of antioxidants and drug-targeting molecules, transcriptomic networks based on a combination of N-acetyl-L-cysteine (NAC) and glutathione were analyzed with predictions (Figures 3(b) and 3(c)). NAC targeted BCL2 apoptosis regulator (*BCL2*), fibroblast growth factor receptor 2 (*FGFR2*), and CD36 molecule (*CD36*), which were downregulated in the transcriptomic network (Figure 3(b)). Moreover, glutathione targeted BCL2 apoptosis regulator (*BCL2*), fibroblast growth factor receptor 2 (*FGFR2*), angiotensinogen (*AGT*), and albumin (*ALB*), which were downregulated in the transcriptomic network (Figure 3(c)). In silico prediction of the transcriptomic network indicated that NAC is more effective for the reduction of lipid peroxidation than glutathione. With NAC treatment, the lipid peroxidation level was suppressed, and the loss of cell viability was also slightly decreased. For GSH, the increase in the former function was less than that in the control, and the latter function showed a similar tendency to that of NAC-treated hBM-MSCs. Moreover, one study showed that antioxidants inhibit ROS production and help adipose tissue-derived mesenchymal stem cells maintain their stemness and ability to differentiate multidirectionally [145]. Taken together, it is highly possible that the quality and freshness of cells can be enhanced in the presence of antioxidants. Further studies require wet lab experiments to verify this in silico prediction.

There have been holistic advancements in the quantification of omics, including genomics, transcriptomics, small RNA-omics, proteomics, metagenomics, phenomics, and metabolomics [147]. Several layers of investigations, including those of the proteome, metabolome, transcriptome, genome, and epigenome, have resulted in the heterogeneity and high dimensionality of biological data. Hence, omics data could be combined in a sequential or simultaneous way to decipher the interplay of molecules. Recently, several studies have shown that the combined omics data lead to a better understanding of the biological system [148–151]. Shin et al. reported that NAC, a ROS scavenger, can protect hBM-MSCs from lipid peroxidation by integrating transcriptomics and metabolomics with amino acid profiling. Thus, they emphasized that multiomic analysis, such as the integration of transcriptomics and metabolomics (metabotranscriptomics), can be one strategy to overcome the limitations of conventional analyses of the condition of hBM-MSCs [23]. Moreover, studies on the application of miRNA to neurological disease have been reported based on posttranscriptional gene repression or the degradation properties of various miRNAs in multiple targets [152, 153]. Metabotranscriptomics integrated with small RNA-omic analysis might provide a clear rationale with respect to the importance of maintaining the quality and freshness of hBM-MSCs before clinical use.

Computational approaches, like machine learning, aid in handling vast amounts of generated data, such as omic big data. Machine learning can be classified into three types as follows: (i) supervised, (ii) unsupervised, and (iii) semisupervised or reinforcement. Among them, the unsupervised machine learning approach learns patterns from the unlabeled dataset and groups them based on data resemblance [154]. Especially, unsupervised methods of multivariate statistical analysis include principal component analysis, self-organizing maps, hierarchical clustering, and *K*-means. These methods reduce the dimensionality of data and can be used to visualize clusters (classifications) based on data similarity among samples. Particularly, *K*-means clustering is a traditional approach in unsupervised machine learning that can handle huge datasets to generate globular-shaped tight clusters using less computational time. Therefore, compared to other machine learning algorithms, *K*-means clustering is a very useful algorithm for the integration of omics data.

The integration of omics, advanced machine learning algorithms, and bioinformatic tools enable researchers to analyze feasible studies on the quality and freshness of hBM-MSCs based on the accurate discrimination of changes in the levels of omics data and the in silico prediction of phenomena using integrated transcriptomics and metabolomics. Therefore, to improve the efficacy of stem cell therapy with respect to the quality and freshness of hBM-MSCs, studies on comprehensive multiomic analysis (big data) and proper machine learning are required to analyze correlations within data. Moreover, in silico prediction is highly recommended, rather than a targeted approach, according to the complexity of dissociated hBM-MSCs.

In the review, we focused on describing strategies to improve the quality and freshness of hBM-MSCs for the treatment of neurological diseases. However, these factors are also affected by additional variables such as elevated temperature, high ionic strength, and nonoptimal substrate composition of the storage solution [139]. For example, storage temperature is an important factor affecting the quality of stored stem cells. Several temperature conditions were evaluated such as cold storage (4°C), low temperature (16-20°C), room temperature (25°C), physiological temperature (37°C), and cryopreservation [-20°C, -80°C, and -196°C (liquid nitrogen)] [139, 155]. There were advantages and disadvantages concerning the impact on storable time, differentiation capacity, viability, and protein secretion at the various temperatures [155].

Additionally, cryopreservation enables the storage of MSCs for a comparably longer period (over a month) than nonfreezing storage (one week). However, cryoprotective agents such as small (e.g., dimethyl sulfoxide, glycerol, ethylene glycol, and propylene glycol) and high molecular weight (e.g., sugars, polyvinylpyrrolidone, and hydroxyethyl starch) penetrating and nonpenetrating agents, respectively, are required to preserve the cellular functional and structural integrity [156]. Cryoprotective agents such as serum and serum alternatives have been used with dimethyl sulfoxide [156]. Moreover, the use of cell containers, impact of the freezing and thawing process, and the elution of cryoprotective agents should be considered during cryopreservation [156]. Free radical scavengers, ion chelators, protease inhibitors, and Rho-kinase inhibitors (Pinacidil, FDA-approved) have been used for the prevention of cryopreservation-induced cell death [157, 158]. However, the duration of storage time was the same in vitro, with improved therapeutic effects of hBM-MSCs observed using earlier passage (passage 2) than later passaged cells (passage 6) after *intravenous* administration of ex vivo cultured hBM-MSCs in a rat model for ischemic stroke [159]. Therefore, further studies are required to evaluate the quality and freshness of stored hBM-MSCs before use in human clinical trials.

## 4. Conclusion and Future Perspectives

Here, we reviewed hBM-MSCs, their application to neurological diseases, and improvements in the quality and freshness of these cells based on integrated omics after disassociation from the culture dish for stem cell therapy. As classical approaches are limited in terms of analyzing the quality and freshness of dissociated hBM-MSCs, the omics and machine learning approaches provide indepth and comprehensive information on the characteristics of the quality and freshness of dissociated hBM-MSCs. Therefore, further studies are needed regarding the integrated multiomic analysis, including genomics, transcriptomics, small RNA-omics, proteomics, phenomics, and metabolomics, in various hBM-MSCs conditions. Since multiomic is big data, application of machine learning algorithms for the multiomic analysis of hBM-MSCs will be one of the approaches for accurate discrimination and in silico prediction of the biological phenomena. Thus, these approaches will be helpful to analyze cellular changes of dissociated hBM-MSCs in the various conditions and improve their quality and freshness for successful stem cell therapy in neurological diseases.

## Figures and Tables

**Table 1:** Genes related to the transcriptomic network of the quality and freshness of starved human bone marrow-derived mesenchymal stem cells (hBM-MSCs).

Entrez gene name	Symbol	Affymetrix ID	Location	Signal (fold change)^a^	
				6 h	12 h
Phosphatidylinositol-4,5-bisphosphate 3-kinase catalytic subunit gamma	*PIK3CG*	206370_at	Cytoplasm	-11.70	-22.42
erbb2 interacting protein	*ERBIN*	232896_at	Cytoplasm	-1.10	-19.71
Solute carrier family 25 member 27	*SLC25A27*	230624_at	Cytoplasm	-9.20	-12.39
BCL2 apoptosis regulator	*BCL2*	207005_s_at	Cytoplasm	-7.73	-9.99
Peroxiredoxin 3	*PRDX3*	209766_at	Cytoplasm	1.85	-9.27
NADPH oxidase 5	*NOX5*	1553023_a_at	Cytoplasm	1.70	11.82
Neutrophil cytosolic factor 4	*NCF4*	205147_x_at	Cytoplasm	-1.19	12.5
Protein kinase AMP-activated catalytic subunit alpha 2	*PRKAA2*	238441_at	Cytoplasm	12.61	22.26
Phosphatase and tensin homolog	*PTEN*	242622_x_at	Cytoplasm	3.62	54.26
Angiotensinogen	*AGT*	202834_at	Extracellular Space	1.04	-27.13
TNF superfamily member 14	*TNFSF14*	233935_at	Extracellular Space	-1.19	-23.12
Albumin	*ALB*	211298_s_at	Extracellular Space	8.07	-20.19
Alpha-microglobulin/bikunin precursor	*AMBP*	214425_at	Extracellular Space	-4.55	-10.51
Adiponectin, C1Q, and collagen domain containing	*ADIPOQ*	207175_at	Extracellular Space	-3.15	-10.1
Serpin family B member 5	*SERPINB5*	1555551_at	Extracellular Space	6.58	10.19
Insulin-like growth factor 1	*IGF1*	209542_x_at	Extracellular Space	8.20	17.32
von Hippel-Lindau tumor suppressor	*VHL*	203844_at	Nucleus	-1.33	-37.9
MacroH2A.1 histone	*MACROH2A1*	1558779_at	Nucleus	1.76	12.75
Integrin subunit beta 1	*ITGB1*	215878_at	Plasma Membrane	12.40	-36.23
Fibroblast growth factor receptor 2	*FGFR2*	211400_at	Plasma Membrane	4.66	-15.37
Low-density lipoprotein receptor	*LDLR*	217103_at	Plasma Membrane	-12.50	-11.9
Glutamate metabotropic receptor 5	*GRM5*	207235_s_at	Plasma Membrane	-10.02	-10.17
CD36 molecule	*CD36*	242197_x_at	Plasma Membrane	3.27	-9.46
Presenilin 1	*PSEN1*	1559206_at	Plasma Membrane	-2.64	9.57
Fc fragment of IgG receptor IIa	*FCGR2A*	203561_at	Plasma Membrane	13.11	13.69
Toll-like receptor 7	*TLR7*	220146_at	Plasma Membrane	2.10	24.72
Prostaglandin E receptor 3	*PTGER3*	210375_at	Plasma Membrane	-17.22	27.73

^a^Normalized ratio of fold change of the signal at 6 and 12 h of storage, relative to the corresponding signal of the control group.

## Data Availability

The data used to support the findings are included within the article.
